# Identification and ranking of environmental threats with ecosystem vulnerability distributions

**DOI:** 10.1038/s41598-017-09573-8

**Published:** 2017-08-24

**Authors:** Michiel C. Zijp, Mark A. J. Huijbregts, Aafke M. Schipper, Christian Mulder, Leo Posthuma

**Affiliations:** 1 Department of Sustainability, Environment and Health, National Institute for Public Health and the Environment, P.O. Box 1, 3720 BA Bilthoven, The Netherlands; 2 Department of Environmental Science, Radboud University Nijmegen, P.O. Box 9010, 6500 GL Nijmegen, The Netherlands

## Abstract

Responses of ecosystems to human-induced stress vary in space and time, because both stressors and ecosystem vulnerabilities vary in space and time. Presently, ecosystem impact assessments mainly take into account variation in stressors, without considering variation in ecosystem vulnerability. We developed a method to address ecosystem vulnerability variation by quantifying ecosystem vulnerability distributions (EVDs) based on monitoring data of local species compositions and environmental conditions. The method incorporates spatial variation of both abiotic and biotic variables to quantify variation in responses among species and ecosystems. We show that EVDs can be derived based on a selection of locations, existing monitoring data and a selected impact boundary, and can be used in stressor identification and ranking for a region. A case study on Ohio’s freshwater ecosystems, with freshwater fish as target species group, showed that physical habitat impairment and nutrient loads ranked highest as current stressors, with species losses higher than 5% for at least 6% of the locations. EVDs complement existing approaches of stressor assessment and management, which typically account only for variability in stressors, by accounting for variation in the vulnerability of the responding ecosystems.

## Introduction

Ecosystems are under influence of human-induced stress all over the world^1^. Some authors even speak of an ‘anthropogenic biosphere’^2^. The diagnosis of environmental impacts, including identification and ranking of stressors, is of key importance for protective and/or restorative management. The influence of human stressors on ecosystems depends not only on the types and magnitudes of the stressors, but also on the vulnerability of the impacted ecosystems. Hence, to reach policy goals regarding sustainable management of our environment, such as the UN development goals at the global scale^3^ or good ecological status goals for ecosystems at the regional scale (e.g. ref. 4,) knowledge is required on both the types and magnitudes of the man-made stresses posed on ecosystems as well as on ecosystem vulnerabilities^5, 6^. Ecosystem vulnerability varies in space and time, because it depends on the interplay between environmental conditions and species assemblages, which are both location-specific features^7–10^. In environmental assessments and management, however, variation in ecosystem vulnerability is commonly ignored. For example, regulations on the production and use of chemicals are often based on a uniform exposure (concentration) boundary, which is derived from the responses of a small selection of individual species exposed to single compounds under laboratory conditions^11, 12^. Neglecting variation of ecosystem vulnerability to stressors leads, however, to either under-protection^13, 14^ or over-protection^15^.

In this study we designed a method to characterize the variability of ecosystem vulnerabilities over a large region, and we refer to the resulting distribution as the Ecosystem Vulnerability Distribution (EVD). The EVD quantifies the range of ecosystem vulnerabilities in a region for a stressor based on the environmental responses of a set of species assemblages as observed in a selection of reference sites. This is similar to the derivation and use of species sensitivity distribution (SSD) models for describing across-species variation in sensitivity for a toxic compound^16^. For chemicals, the overlay of the SSD with the stressor exposure distribution for a region has been proposed as a measure of ecological risk, and has – via groundbreaking studies on atrazine risks across sites in North America^17^– had global influences on environmental protection, assessment and management regarding plant protection products^18^. EVDs can be applied likewise, but now for stressor identification and ranking across multiple potentially relevant stressors based on vulnerability distributions described on the basis of actual monitoring data on species assemblages. Thereby, the approach can assist in bridging the current gap between assessments of the impacts of chemical mixtures and those of other stressors^19^.

**Figure 1:**
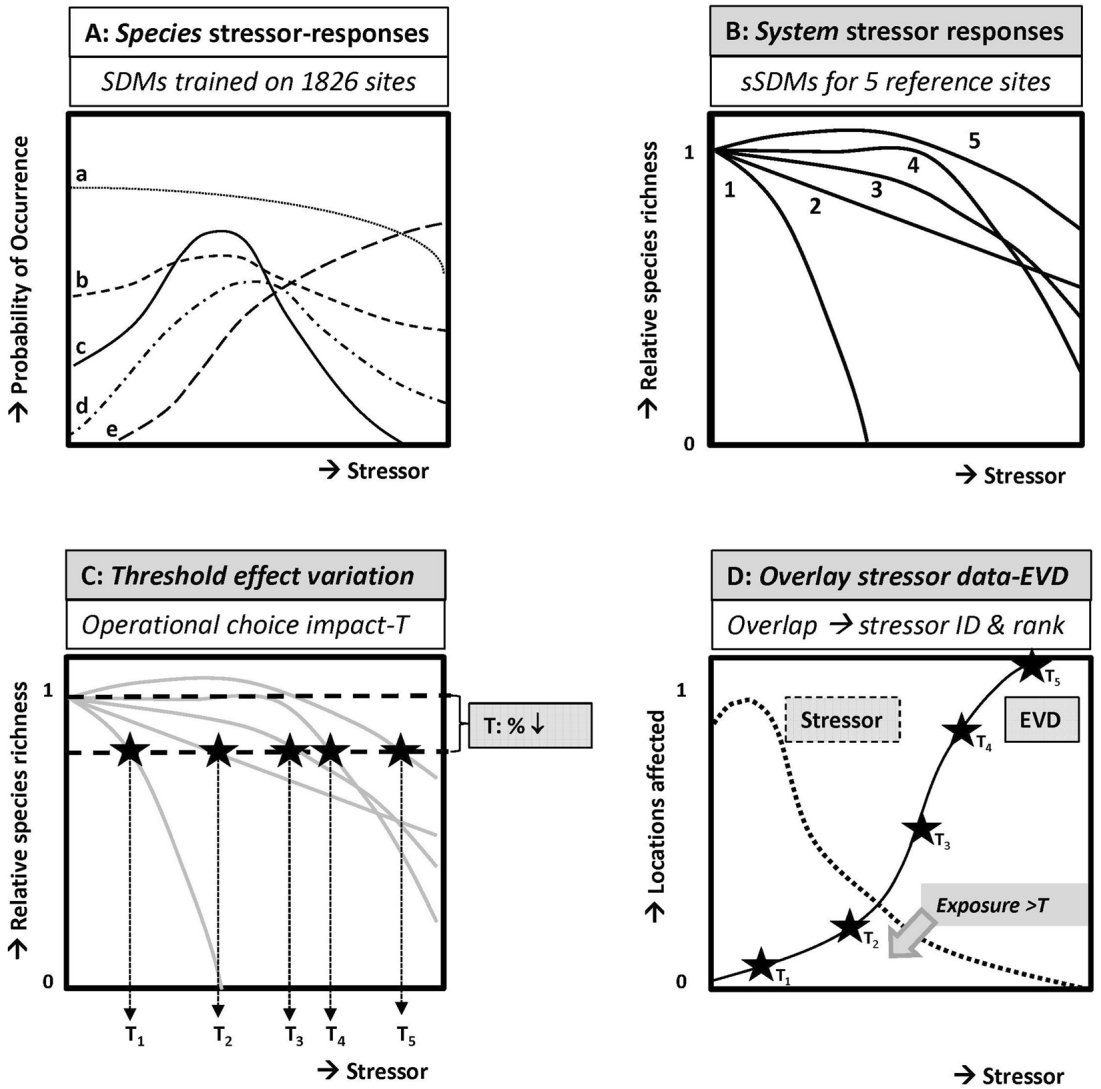
Conceptual diagrams of the derivation and use of ecosystem vulnerability distributions (EVDs), illustrated with five sites (i = 1–5) as reference sites. (**A**) Species distribution models (SDMs) are used to describe the probability of occurrence of species (**a**–**e**) as a function of a stressor variable. (**B**) The probabilities of occurrences are recalculated towards relative species richness (RSR) for each of the five reference sites (defined as Y = 1 under prevailing conditions) and subsequently the site-specific responses of this RSR to a stressor are derived; (**C**) when a selected magnitude for loss of species richness is selected as threshold (T) for impacts, the stressor-response curves yield a set of site-specific threshold stress level, T_1_ up till T_5_; (**D**) the values of T_1_ up till T_5_ are interpreted as a distribution of vulnerabilities, and this panel thus summarizes the Ecosystem Vulnerability Distribution for a stressor. Panel D also shows the utility of the EVD for stressor identification and ranking. An overlay of the stressor level distribution for the studied region and its EVD reveals if an environmental variable is considered to act as a stressor in the region (stressor identification), and (in a comparison across stressors) the degree of overlay relates to stressor ranking.

The EVD for a region and a selected stressor is derived in various steps, conceptually depicted in Fig. 1A–D. First, based on monitoring data of species assemblages and environmental conditions, a selection is made of reference sites that are considered representative for the ecosystems in the region of interest^20^. For the species observed at those reference sites species distribution models (SDMs) are established using monitoring data of the whole region. The SDMs enable quantification of the probability of occurrence of each of the species in relation to the set of environmental variables measured at any site. Next, the SDMs are used to quantify the stressor-response relationships of the species assemblages of each of the reference sites to a selection of the environmental variables, i.e., the stressors. This is done by varying the stressor of interest across its range while fixing all other environmental variables at their site-specific values (Panel A in Fig. 1). The resulting probability of occurrence outcomes are aggregated over all species in the assemblage specific to each reference site, resulting in site-specific responses of species richness to the stressor of concern, relative to the species richness corresponding with the actual conditions at the reference site (Fig. 1 panel B). Subsequently, a threshold (T) is selected, whereby T represents a specific magnitude of loss of species richness, which is then used to quantify the stressor levels that result in this loss at the reference sites (Fig. 1 panel C). The variation of these stressor levels over the reference sites yields the EVD for that stressor in the region of interest (Fig. 1 panel D, the EVD). The EVD operationally summarizes the observed variability of responses of ecosystems to the application of a stressor, and it can be interpreted as the description of vulnerabilities of *all* sites in a region based on the information collected using the selected reference sites. Utilizing the principle of overlaying this distribution with the distribution of the associated stressor variable for the whole region implies stressor identification (when there is overlap) and ranking (when the overlap increases, the influence of the stressor variable on local ecosystems likely increases, Fig. 1 Panel D). Further details of the steps are explained in the method section.

We applied the method, and evaluated the stressor identification and ranking outputs, in a case study with assemblages of freshwater fish species across Ohio (USA). Freshwater ecosystems are amongst the most intense human-impacted habitats^21^. Ohio is a state with a wide variety of human activities and environmental conditions.

The case study started with a collection of statewide biomonitoring data for Ohio freshwater systems (n = 1,826), the selection of a subset of 18 sites that are in good and stable ecological condition while representing the region under consideration (i.e., the reference sites^22^) and the derivation of species-specific SDMs for the occurrence of fish species at those sites. The environmental variables taken into account in the fitting of the SDMs were drainage area, water acidity (pH), mixture toxic pressure, nutrient loads, hardness, conductivity and habitat quality. The latter was quantified by the Qualitative Habitat Evaluation Index (QHEI^23^), a measure for the physical-habitat quality for fish, developed for running waters in Ohio that ranges from 0 (poorest quality) to 100 (maximum quality). Mixture toxic pressure was expressed as the multi-substance Potentially Affected Fraction of species (msPAF_EC50_) derived from the 50% effect concentrations of metals, ammonia and nitrite^24, 25^. Further methodological details are explained in the method section.

## Results and Discussion

### Species-specific environmental responses

In general, the SDMs showed a reasonable to good fit, with 90% of the models exhibiting high explained occurrence variation (Area Under Curve [AUC] values >0.7, Table S3)^26, 27^. The combinations and values of the regression coefficients of the SDMs varied widely across the species (Fig. S1 and Table S3), highlighting that different fish species respond differently to the same environmental variables. This observation already implies that the ecosystem vulnerability must differ between sites inhabited by different species assemblages. In general, drainage area and QHEI were mostly characterized by positive responses (Fig. S1, grey bars), whereas the median of the coefficients for TP was the smallest, indicating a negative influence between this variable and the probability of occurrence of most fish species. Further, the 5^th^ and 95^th^ percentiles of the estimated SDM-coefficients across the species were negative and positive for all the environmental variables, respectively (Fig. S1). This means that all environmental variables may induce positive as well as negative responses, depending on the stressor level and species.

### Assemblage-specific responses to stress

**Figure 2:**
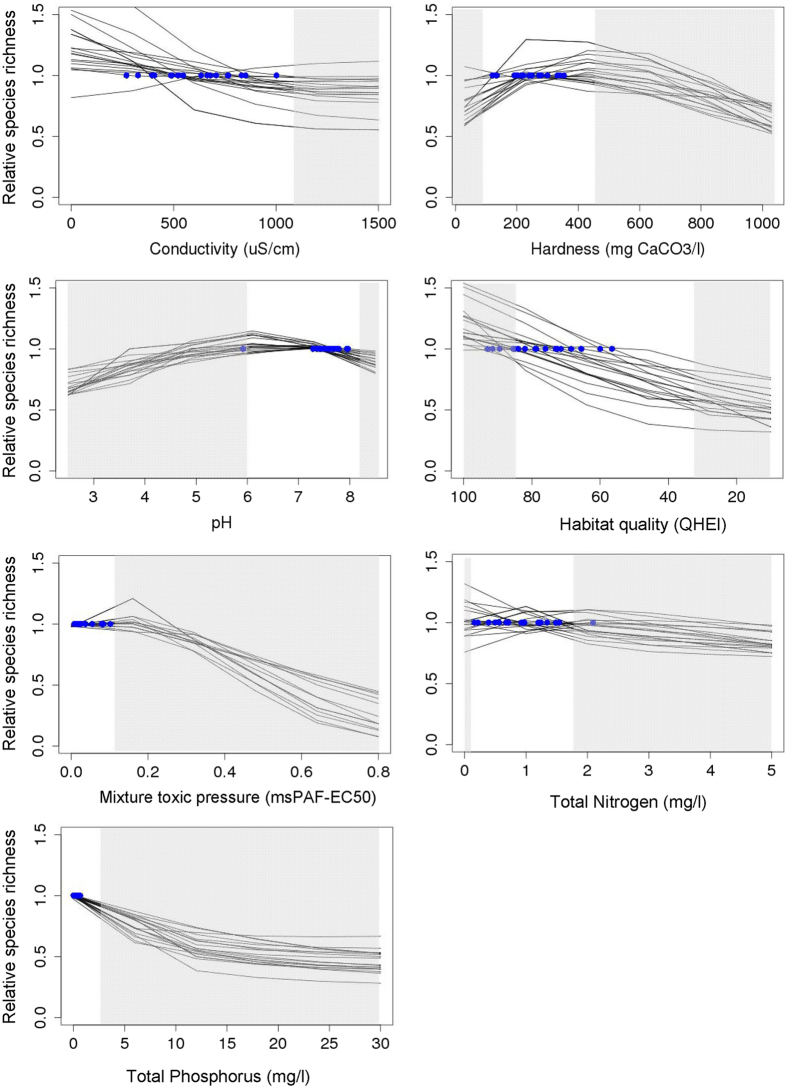
Modelled stressor-response relationships for Ohio fish species assemblages for the selected 18 reference sites, showing the response of relative species richness (RSR) to conductivity, hardness, pH, QHEI, mixture toxic pressure, Total N and Total P. Values of RSR higher than 1.0 indicate an increase compared to the situation observed at the reference site and *vice versa*. The ‘points of departure’–RSR at reference conditions–are shown as blue dots. The white areas represent 90% of the stressor-level variability in the monitoring data of Ohio.

The SDMs were used to quantify the probability of occurrence of reference sites species. The site-specific stressor-response curves derived for the reference sites resulted in three key observations. First, assemblages of fish species at different reference sites responded differently to imposed stress (Fig. 2), whereby the ‘points of departure’ (the conditions at the reference sites, plotted as dots in Fig. 2) illustrate the variability of the natural conditions for the set of reference sites. Second, different stressors yielded stressor-response curves of different shapes. Changes in pH resulted in bell-shaped curves, indicating that both an increase and a decrease of the stress level can cause a decline in relative species richness (RSR). Mixture toxic pressure and hardness showed a skewed optimum: a slightly increasing mixture toxic pressure or hardness yielded (slight) increases of relative species richness, followed by significant reductions at increased stressor values. The response curves for QHEI and TP were approximately linear for most sites. Change in conductivity resulted in only a marginal decline in RSR at 15 of the 18 sites, reflecting canceling out of positive and negative species-specific responses. Change in TN yielded a diversity of responses of RSR, including linear, unimodal and skewed shapes. Third, the response curves did not reveal clear tipping points, hence the available data did not provide a clear basis to identify a natural threshold of impacts, as all species loss curves were gradual^9^. The stressor-response curves were robust to a different selection of species, i.e., response curves did not change when SDMs with AUC < 0.7 were excluded (compare Figs 2 and S2).

### Ecosystem vulnerability distributions (EVDs)

**Figure 3:**
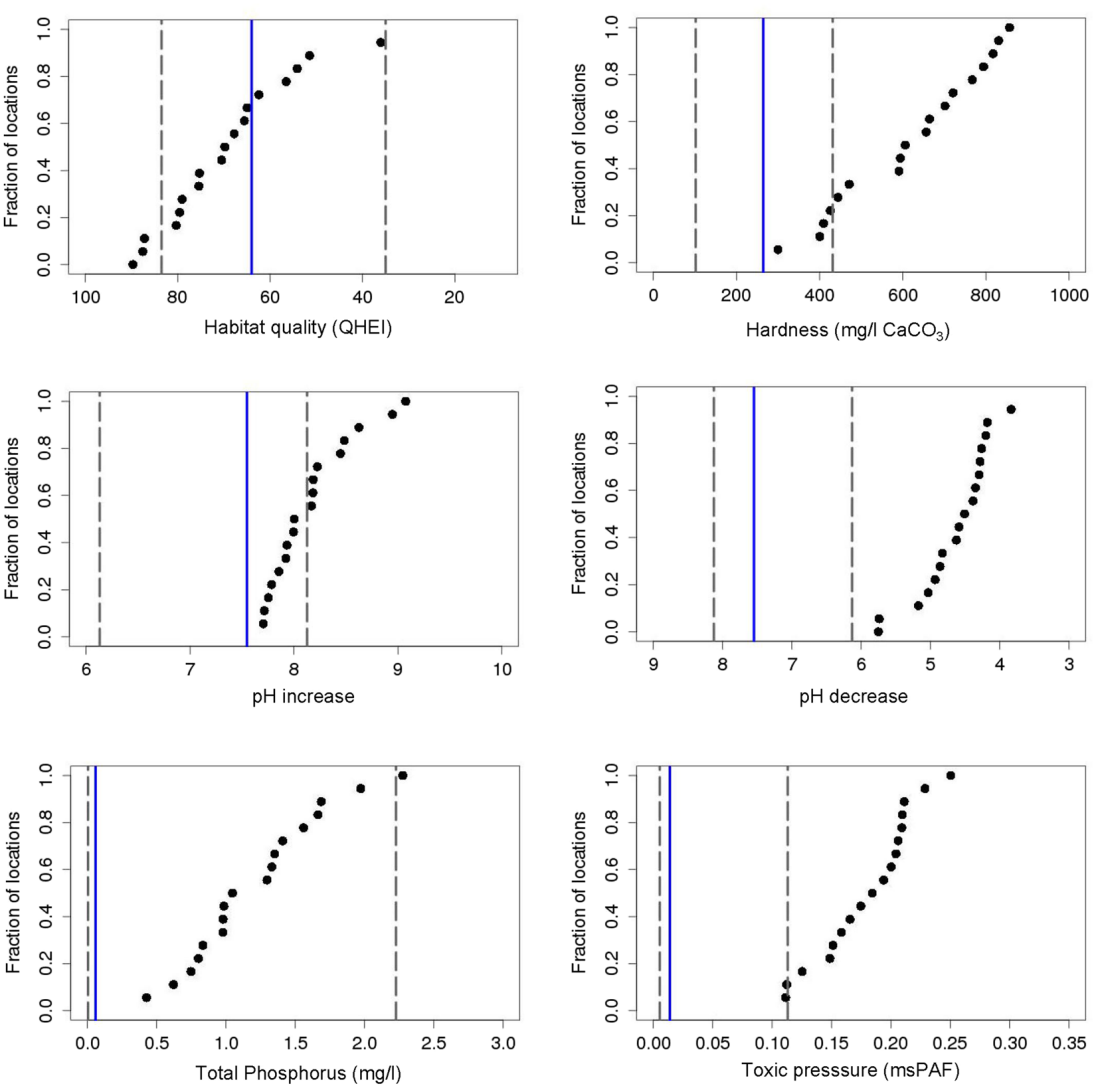
Stressor-specific ecosystem vulnerability distributions (EVDs) for hardness, habitat quality, pH (decrease and increase), mixture toxic pressure and total phosphorus, showing for each stressor the fraction of sites at which the threshold of the selected 5% species loss threshold is exceeded. The median stressor values across Ohio (1,826 sites) are indicated with a vertical blue line. The areas between the two dotted lines contain 90% of the stressor-level variability in the monitoring data.

Predicted assemblage-level responses to stress were indeed specific to sites and stressors (Fig. 3). Because of the low response to conductivity and the complex response to TN, the stressor-specific EVDs were not derived for these two variables. For pH two EVDs were derived, one for increasing and one for decreasing values, as compared to the reference-measured value. In the absence of observed natural tipping points, the EVD was based on a selected impact threshold of 5% loss of species richness. A test was executed to investigate whether the EVD-patterns are robust to selections of impact thresholds other than T = 5% loss, resulting in the observation that vulnerabilities indeed differ across sites, with a right-shifted pattern of the EVDs at higher values of the threshold impact (SI Fig. S3).

The most vulnerable and least vulnerable reference sites, and the pattern of data for the other reference sites, determine the known EVD range. Utilizing the EVD-pattern for stressor identification and ranking by overlaying the stressor distribution with the associated EVD showed that, for example, the mixture toxic pressure level that would induce 5% loss of species richness ranged between 0.11 and 0.25 across the sites, hence differed by more than a factor of two.

### EVD-utility: landscape-level stress identification and ranking

**Figure 4:**
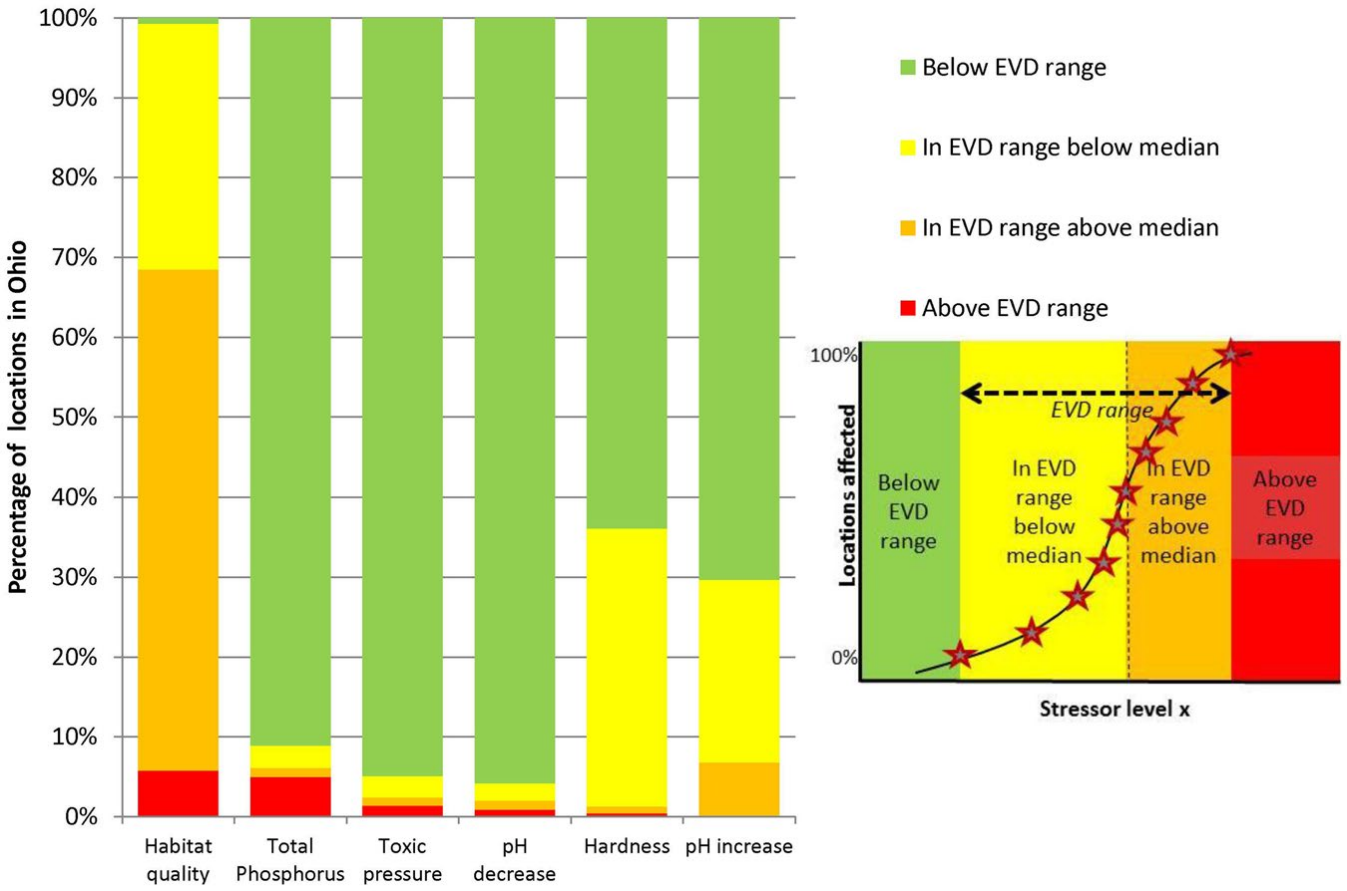
Stressor identification and ranking, resulting from an analysis of the overlays between stressor-specific EVDs and the regional stressor value distributions of the 1,826 monitoring sites in Ohio.

Conclusions on stressor identification and ranking were derived from the overlays of the measured stressor values and the associated EVD, considering the region from which the reference sites were selected and which they aim to represent. The presence and degree of overlay of the EVD-ranges with the ranges of observed stressor values across the 1,826 monitoring sites of Ohio were interpreted as a landscape-level risk indicator and could be used for stressor identification and ranking (Fig. 4), similar to the approach in ecotoxicology where the overlay of exposure- and sensitivity distributions for species yields an aggregated measure of the ecotoxicological risks of a chemical for an area, with proven utility for practical decision support purposes in the management of hazardous chemicals^17, 18, 28^. Note that the present study bridged the problem that water quality managers are often confronted with, i.e. distinct assessments for chemical pollution and other stressors^19^. Furthermore, the results per stressor can also be presented as map, revealing the geographical locations where the sites’ stressor level is below, within or above the derived EVD ranges (see SI Fig. S5), i.e. a geo-referenced stressor map.

Ecological responses in the study region are mainly related to QHEI and TP. The comparison of the EVDs with field observations (Fig. 4) shows that, of all stressors, reduction of QHEI is of the highest concern for the occurrence of fish species. At nearly 6% of the locations the current QHEI results in a species richness reduction higher than the selected threshold of T = 5% (red bar in Fig. 4). Furthermore, 94% of the locations in Ohio are *at risk* of exceeding the T = 5% loss threshold for QHEI. For TP, 91% of the region is not at risk for exceeding the T = 5% species loss. Of the other 9%, 5% is exposed to TP concentrations above the EVD range, implying a higher loss of fish species than T = 5% due to increased levels of TP, and 4% of Ohio is exposed in the range of the EVD stress levels and are thus at risk of more than T = 5% loss of fish species due to increased levels of TP. A confirmation study showed that the obtained stressor identification and ranking results are well in line with those of earlier assessments, as habitat modification and nutrients were identified amongst the five most important stressors for freshwater ecosystems in the recent Integrated Water Quality Monitoring and Assessment Report of Ohio Environmental Protection Agency^29^.

### Methodological robustness

The shape and position of EVDs depend on various methodological characteristics. Firstly, the EVDs may change with species selection. SDMs can be established only for species with enough monitoring observations, so that the method may not represent situations where there are rare or poorly detectable species. Theoretically, common species are usually considered more relevant from an ecosystem functioning perspective^30, 31^, so that such widely-occurring species might be considered more important to be included in the EVD than (co-occurring) rare species. Yet, this implies that other complementary indicators may be needed to complement the stressor analyses from a species conservation perspective^30, 32, 33^. Further, we utilized fish species observed at the reference sites for the derivation of the EVDs. Species that are not currently observed at a reference site but are observed at other sites in the region, may, however, still be able to reach and survive there. To check the possible dependency of the EVD-approach on species selection, we also derived EVDs from an alternative viewpoint, that is, the calculated RSR for all fish species in Ohio for which an SDM could be derived. This provides an EVD_all_species_ per stressor, as if all species are present at all reference sites. Able to include 98 species in this exploration, we found that the EVDs for physical habitat quality, total phosphorus and pH became more steep (see SI Fig. S4). Despite these shifts, the stressor identification and ranking remained the same. Secondly, the EVD may change with a different selection of abiotic variables, as the responses of the species to each of the variables are contingent on their responses to other abiotic factors. A careful selection of relevant abiotic variables and possible confounders is needed to avoid that EVDs are based on spurious relationships. In the case study this was operationalized by selecting variables of ecological relevance, while avoiding multicollinearity by using variance inflation factors (further details in the methods section). Thirdly, a selection of other reference sites will lead to different results. Here we used food web allometry, but in other case studies the selection approach could for example be based on stressor levels and expert judgement. Mulder and Mancinelli^34^ have argued that both reference sites selection and sampling methods matter for the results obtained from ecological modeling, with the former less important than the latter. The influence of these methodological aspects (selection of species, abiotic variables, reference sites and sampling methods) should be considered in the derivation and use of EVDs, and although our stressor identification and ranking results are in line with literature and expert assessments, the effects of methodological issues deserves further investigation. Fourthly, in the absence of observed natural tipping points, we selected T = 5% reduction of the species richness as an impact threshold needed to define the vulnerability distribution. We explored whether this operational choice matters for the key conclusion, and found that a higher impact threshold only resulted in right-shifted EVDs (SI Fig. S4). As a consequence, a change in the selected threshold neither influenced the conclusion that vulnerability is a distributed characteristic nor the stressor ranking. The final interpretation of the overlay at a higher value of T, however, results in the identification of a lower proportion of sites with stressor levels in or above the (right-shifted) EVD-range. Thresholds might also represent a natural ‘breakpoint’ in stressor-response curves, also called tipping points^35^. We earlier applied such a natural threshold definition via exploring the cascading stress level at which an indirect (secondary) species deletion is predicted to occur^22, 36^. Again, the vulnerability of ecosystems was found to be a distributed characteristic. Finally, the method proposed does not yet account for interactions among stressors and among species. The EVD is derived by increasing one stressor in the model while keeping the other stressors at their current value, while biogeochemical processes can be such that a change in one stressor results in change in other stressors. Inclusion of stressor interactions might alter the SDMs, and subsequently the derived EVDs. In addition to abiotic interactions across the stressors, also biotic interactions among the species may occur, in the form of predator-prey relationships or resource competition. The method presented in this paper did not include this. Quantifying inter-species interactions across many fish species is an evolving field with substantial challenges^37^, particularly because trophic relationships among fish species may alter during the natural life history. This would be different for some terrestrial systems, where species may exhibit clear and strong mutual interactions, like between pollinating species and host plant^38^. Yet, the addition of location-specific data on trophic interactions among fish species can be a pathway to improve the SDMs^39^ and may also allow for identification of thresholds based on secondary effects^36^.

### Implications

We showed that ecosystem vulnerabilities for stressors in a region are distributed, irrespective of methodological choices. This implies, amongst others, that exceedance of a protective criterion, such as a regional concentration criterion for a chemical used as protection threshold, will result in dissimilar responses across ecosystems. Furthermore, we showed how EVD models operationalize this implication for the purpose of stressor identification and ranking. This approach can, as example, be used to assess whether the goals of the (European) Water Framework Directive or the (American) Clean Water Act are reached, and if not, which stressors are probably responsible for observed impacts^4^. One of the difficulties in the implementation of these regulations is the meaningful evaluation of variation in stressor levels in relation to the variation in vulnerabilities. The use of pre-set, generic decision criteria in the evaluation of monitoring data may easily lead to Type-1 errors (stressor impacts diagnosed when absent)^40^. The EVD takes this variation into account and could be used as first-tier method for the screening and ranking of potential stressor influences across multiple locations in a larger region. When EVDs are based on sites representing reference conditions, stressor levels in the region lower than the EVD range can be interpreted as identifying sites at which the stressor is not limiting for the good ecological status of a site; levels higher than the EVD range may imply an affected status; and locations with stressor levels within the EVD range are at risk and should be subject to further assessments. The EVD-method thereby also allows for a dynamic assessment of implications of future changes in stressor levels caused by societal developments and/or stressor abatement strategies. This links ideally with the recent call for solution-focused approaches, which are characterized by the early definition of alternative solutions in the assessment process, and the associated need to explore the expected responses of alternative management scenarios^41–43^. Therefore, EVDs can also be used in regional planning activities, deciding on the prohibition or allowance of new activities in the region such as a change in land use or the installation of new wastewater treatment techniques. These types of change in activities have impact on levels of different stressors at the same time (e.g. nutrients, toxic pressure and pH) and the approach in this paper can be used to assess site-specific assemblage responses to changing levels of different stressors simultaneously.

As explained above, the choice of the set of reference sites is important, and commonly the ‘anchor point’ to judge impacts will relate to the policy goals of a good ecological status of all water bodies in a region. However, the method can also be applied when it is based with purpose on deriving the EVDs using a selection of impacted sites. In such an approach, the vulnerability distribution (its position and shape, as well as the overlay with the landscape stressor distribution) is based on a set of sites influenced by human impacts. Overlaying such a ‘stressed-sites’ EVD with the distribution of the stressor levels would provide insights whether stressor levels would be able to *further* affect the species assemblages at such sites, beyond the impacts already present.

Next to stressor identification and ranking, the method allows for defining region-specific impact boundaries, e.g. at the ‘safe side’ (left) of the EVD range or at the stressor level at which a selected minimal percentage of the locations exceeds a selected impact threshold. Such region-specific boundaries could be applied in risk management and when assessing ambient stress via sustainability metrics such as the nitrogen footprint method^44^ and the chemical footprint method^45^.

Finally, in the context of the planetary boundaries concept, Steffen *et al*.^7^ pose that various stressors primarily operate at the regional scale, and thus also cross boundaries at local and regional levels. In those cases aggregation to the planetary level is needed to judge global impacts^7, 46^. Application in this context is a feasible next step in EVD development and use. The EVD approach can be used to explore the fraction of sites that are at risk of exceeding local ecosystem vulnerability for a suite of stressors, given a selected impact threshold or a chosen ‘safe’ boundary below that.

## Methods

### Data selection

Data on the occurrence of the fish species and co-occurring environmental conditions were available for 1,826 sites sampled between 2000 and 2007 across the state of Ohio, USA^24, 47^. When multiple measurements were available per location the median value was used. The ecosystem vulnerability distribution approach requires a subset of sites for which the vulnerability distribution is derived as ‘point of departure’^20^. In this study, the EVD-derivation and use is illustrated by starting with a subset of 18 sites in stable ecological conditions as identified in an earlier study^22^. In that study reference sites were selected that are ecologically in balance according to a judgment of food-web allometry and network metrics [Ref. 22 and references therein]. These 18 reference sites were inhabited by a total of 93 fish species, with an average species richness of 32 (ranging from 12 to 46 species, with one top-predatory species). We selected 84 fish species that were observed at the reference sites and for which at least twenty data points were available in the database, in order to have a minimum number of observations to derive SDMs^48–50^. Environmental variables selected for the SDMs were pH, concentrations of N (total Nitrogen, TN) and P (total Phosphorus, TP), hardness, conductivity, mixture toxic pressure, drainage area and the Qualitative Habitat Evaluation Index (QHEI). These variables were selected because of ecological relevance^22, 24, 47, 51^ while exhibiting low multicollinearity^52^. We operationalized the latter by selecting only predictors with a variance inflation factor (VIF) less than 5^53^. We did this by sequentially removing predictors with VIF > 5 until all remaining predictors had VIF below the threshold. Although some environmental variables contained a few outliers (e.g. 1.9 for pH, or 75 mg/l for TP), the data were distributed according to the patterns expected for a region characterized by a variety of natural background conditions and forms and intensities of human activities (Tables S1 and S2). Notably, two reference sites had a QHEI lower than the median for all sites (values: 56 and 59, while QHEI_max_ is 100; Table S2). Apparently, these assemblages are ecologically in balance while the physical habitat scores are less than optimal according to the QHEI index.

### Species Distribution Models

We obtained the SDMs with logistic regression (logit link and binomial error distribution) to link the species observations (presence-absence) to the environmental variables, thereby including quadratic terms to account for non-linear responses^53^. The general form of the fitted models is Y = β_0_ + β_1_S_1_ + β_1_′ S_1_^2^ + β_2_S_2_ + β_2_′ S_2_^2^ + β_n_S_n_ + β_n_′ S_n_^2^, with Y = probability of occurrence of a species, S_n_ = observed monitoring value for stressors 1-n, β_1−n_ and β′_1−n_ are the estimated SDM-coefficients for linear and quadratic terms, respectively and β_0_ is the intercept. Environmental variables were standardized prior to model derivation. Per species, all possible combinations of predictors were tried using the MuMin package in R^54^, whereby a quadratic term was only allowed if the corresponding linear term was also included. Resulting species-specific models were ranked according to the Akaike’s Information Criterion (AIC). Per species, the weighted average regression coefficients were calculated with the MuMin package, based on the set of models within two AIC units from the model with the lowest AIC. Finally, accuracy of the resulting SDMs was evaluated based on the Area Under the Curve (AUC) of the receiver-operating characteristics (ROC) plots using the R package PresenceAbsence^55^.

### Ecosystem Vulnerability

The fitted SDMs were used to derive stressor-specific response relationships for each reference site, based on the species occurring and the observed environmental conditions at the reference sites. This was done by changing the level of the stressor of concern within the range of observed values in the region–leaving out extreme outliers–while maintaining the other stressors at their observed location-specific values, quantifying the corresponding probability of occurrence of each fish species occurring at that site with the fitted SDM, and then aggregating the probabilities of occurrence over the species. This yields relationships between the value of a stressor and the change in species richness per reference site. Non-disturbed reference conditions were thereby normalized, defining the relative species richness via RSR = Y = 1.

### Environmental Vulnerability Distributions

EVDs were derived per stressor by combining the relative species richness stressor-response relationships with a selected impact threshold (T). This was done for all environmental variables except drainage area, which was not considered a stressor, but as a predictor that describes natural variation amongst the reference sites. The threshold would ideally be based on scientifically derived ‘tipping points’^35^. When the stressor-response patterns do not show these sudden responses, a user-defined impact judgment threshold T needs to be defined. Here we defined this impact threshold as the stressor level at which the predicted species richness, as derived from the stacked SDMs, is 5% lower than the species richness in reference conditions. This yields a range of the critical stress levels across the reference sites that define the distribution of vulnerabilities across reference sites. Subsequently, the EVD–when based on a selection of sites that represents the variability of the natural, minimally disturbed conditions of a region–is assumed to represent the quantitative distribution of ecosystem vulnerabilities of all sites in the region. The overlays of the distributions of stressor values for the 1,826 sites and their EVDs provide insights in the identities and the ranking of the stressors potentially affecting the ecosystems in the region.

## Electronic supplementary material

Supplementary information
