# Supplementary material for: Identification and ranking of environmental threats with ecosystem vulnerability distributions
Source: Sci Rep. 2017 Aug 24;7:9298. doi: 10.1038/s41598-017-09573-8 (PMC5571148; doi:10.1038/s41598-017-09573-8)
Supplement: Supplementary file 1 — Supplementary information [file 41598_2017_9573_MOESM1_ESM.pdf]

Supporting Information

Title: Identification and ranking of environmental threats with ecosystem vulnerability distributions

Authors: Michiel C. Zijp[a,b, 1], Mark A. J. Huijbregts[b], Aafke M. Schipper[b], Christian Mulder[a] and Leo Posthuma[a, b]

[a] Department of Sustainability, Environment and Health, National Institute for Public Health and the Environment, P.O. Box 1, 3720 BA Bilthoven, The Netherlands
[b] Department of Environmental Science, Radboud University Nijmegen , P.O. Box 9010, 6500 GL Nijmegen, The Netherlands

Table S1. Distribution characteristics of environmental variables (predictors) and species richness of the fish assemblages in the Ohio biomonitoring database (n = 1,826).

**Predictor**	**Mean**	**StDev**	**Min**	**5%**	**25%**	**Median**	**75%**	**95%**	**Max**
Conductivity (µS/cm)	661	303	101	272	475	642	783	1116	4116
Drainage area(km^2)	111	112	86	86	87	88	96	198	1931
Hardness (mg $CaCO_3$/l)	268	121	32	102	190	265	334	431	1431
pH (-)	7.43	0.73	1.92	6.13	7.33	7.55	7.76	8.13	8.68
QHEI (-)	62	15	16	35	54	64	73	84	101
TN (mg/l)	0.82	0.89	0.11	0.16	0.41	0.67	0.99	1.88	25.00
Toxic pressure, $msPAF_{EC50}$ (-)	0.03	0.06	0.00	0.01	0.01	0.01	0.03	0.11	0.72
TP (mg/l)	0.96	5.79	0.00	0.00	0.03	0.06	0.13	2.23	74.44
**Fish assemblage**									
Species richness (-)	18	8	1	6	12	17	23	34	50

Table S2. Distribution characteristics of environmental variables (predictors) and species richness of the fish assemblages for the selected reference locations in Ohio (n = 18).

**Predictor**	**Mean**	**StDev**	**Min**	**5%**	**25%**	**Median**	**75%**	**95%**	**Max**
Conductivity (µs/cm)	604	197	270	318	488	590	751	874	1002
Drainage area (km^2)	194	103	88	100	116	158	214	363	449
Hardness (mg $CaCO_3$/l)	234	69	121	130	201	219	278	343	353
pH (-)	7.52	0.46	5.92	7.08	7.41	7.54	7.73	7.99	8.15
QHEI (-)	78	11	56	59	72	79	85	92	93
TN (mg/l)	0.94	0.50	0.16	0.21	0.59	0.94	1.23	1.62	2.09
Toxic pressure, $msPAF_{EC50}$ (-)	0.03	0.03	0.01	0.01	0.02	0.02	0.03	0.09	0.10
TP (mg/l)	0.16	0.19	0.00	0.02	0.04	0.10	0.20	0.59	0.65
**Fish assemblage**									
Species richness(-)	32	12	12	13	25	33	42	46	46

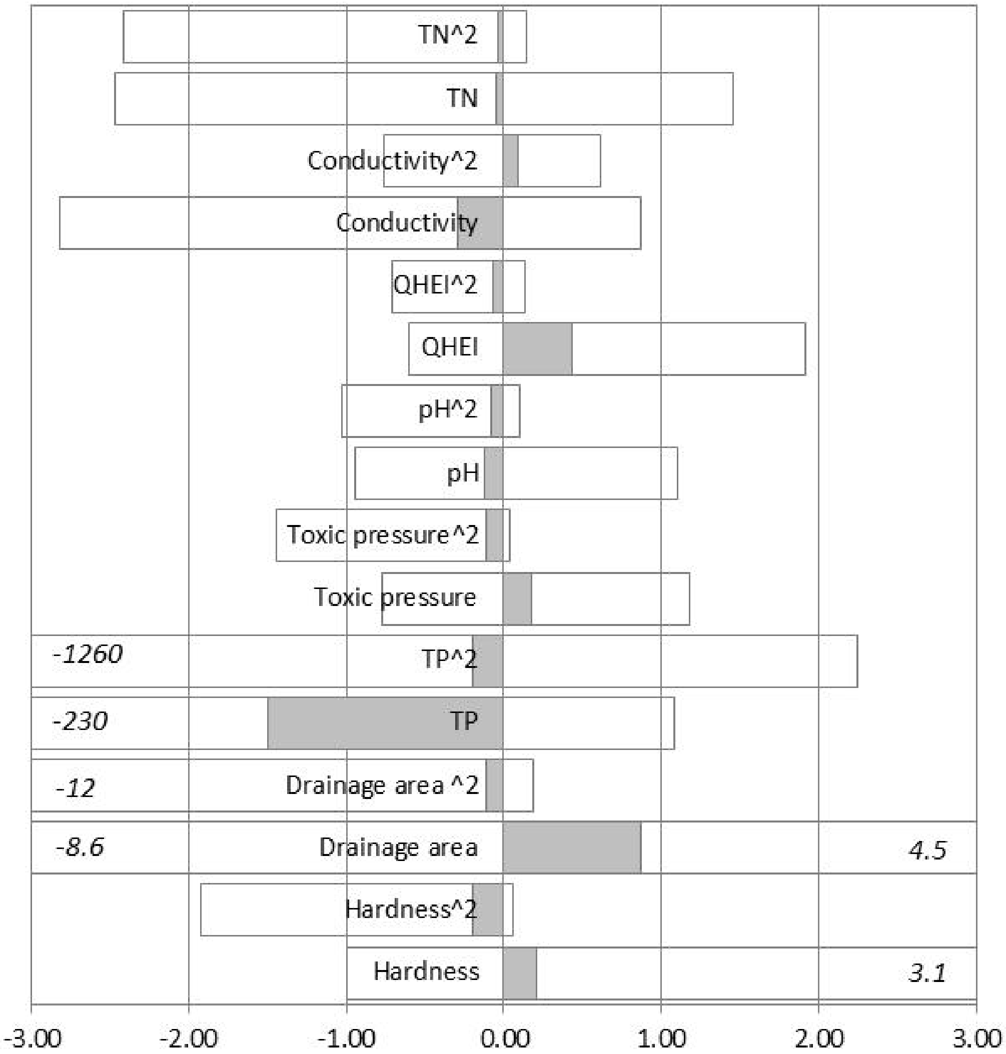

Fig S1. Variability of the standardized regression coefficients for the different environmental variables of the SDMs across the 84 fish species. The grey bar is the median and the bars on the left and right respectively the 5th and the 95th percentile of the coefficients across the species set. Values for the 5th and 95th percentiles that exceeded the figure bounds where cut off and shown as label in the bar.

Table S3. Coefficients and Area Under the Curve (AUC) values of the species distribution models included in this study. msPAF = mixture toxic pressure (at EC50-level), TN = total Nitrogen, TP = total Phosphorus, Cond = Conductivity, QHEI = Qualitative Habitat Evaluation Index, DA = Drainage Area, NA = Not Applicable.

Species names	Intercept	msPAF	$msPAF^2$	pH	pH^2	TN	TN^2	TP	TP^2	$CaCO_3$	$CaCO_3{}^2$	Cond	$Cond^2$	QHEI	$QHEI^2$	DA	DA^2	AUC
Ambloplites rupestris	-0.08	-0.17	-0.02	-0.38	-0.07	0.11	-0.06	-1.22	0.08	0.70	-0.15	-0.86	0.12	0.65	-0.03	1.70	-0.13	0.78
Ameiurus natalis	0.26	0.31	-0.07	-0.14	-0.03	0.06	NA	0.02	NA	-0.23	0.00	0.38	-0.04	-0.02	0.08	0.33	-0.46	0.61
Ammocrypta pellucida	-28.28	1.66	-0.78	1.10	0.26	-2.49	-2.43	-166.68	-2476.25	1.49	0.02	-2.20	-0.38	0.09	-0.68	2.20	-0.13	0.55
Aplodinotus grunniens	-5.49	0.14	NA	-0.51	-0.12	-1.11	0.21	-59.00	-300.20	-0.25	-0.64	-0.11	NA	0.92	0.03	1.83	-0.10	0.93
Campostoma anomalum	1.51	-0.30	-0.03	0.31	0.00	-0.36	0.08	-0.02	NA	0.74	-0.11	-0.72	0.11	0.77	0.12	-1.20	0.07	0.75
Carpiodes carpio	-2.61	0.69	-0.21	-0.20	NA	-0.64	-0.96	-17.82	-184.41	-0.73	-1.14	-0.45	NA	1.03	-0.07	0.98	-0.07	0.91
Carpiodes cyprinus	-2.49	0.27	-0.13	-0.03	NA	0.07	NA	-0.62	-0.35	0.88	-0.58	-1.12	0.35	0.33	0.04	2.52	-0.14	0.88
Carpiodes velifer	-5.91	-0.12	NA	-0.36	0.03	-1.75	-0.37	-55.25	-346.22	-0.09	-0.93	-0.26	NA	1.23	-0.08	0.84	-0.04	0.93
Catostomus commersonii	2.07	-0.41	0.00	0.35	0.04	0.72	-0.03	0.11	0.04	0.10	0.03	-0.06	NA	0.20	-0.15	-1.73	0.09	0.75
Cottus bairdii	-2.11	-0.67	-0.13	0.09	NA	0.14	-0.03	-5.78	-1.56	0.83	-0.06	-0.92	0.08	0.75	-0.06	-1.50	0.08	0.76
Cyprinella spiloptera	0.48	0.26	-0.06	-0.22	-0.06	-0.07	-0.08	-1.16	0.08	0.25	-0.12	-0.30	0.11	0.25	0.12	9.41	-0.57	0.84
Cyprinella whipplei	-4.24	-0.29	NA	-0.38	-0.50	-2.46	0.20	-10.48	-215.79	-0.78	-2.07	-1.24	0.70	4.03	-1.46	4.04	-0.97	0.98
Cyprinus carpio	-0.77	0.15	-0.04	0.09	-0.03	0.52	-0.02	-1.63	0.11	-0.05	NA	0.37	-0.09	-0.10	0.10	3.94	-0.23	0.81
Dorosoma cepedianum	-1.71	0.37	-0.10	-0.47	-0.12	0.65	-0.27	-1.72	0.14	-0.10	NA	-0.02	NA	0.32	0.00	2.49	-0.14	0.83
Erimystax dissimilis	-49.45	-2.16	-4.53	-1.39	-0.49	-1.11	-8.47	-865.58	-3180.85	3.74	-2.28	-3.75	0.91	4.34	-1.10	0.66	-0.05	0.98
Erimystax x-punctatus	-6.05	1.65	-0.66	0.50	-1.62	-1.98	0.07	-3.37	NA	0.20	NA	0.07	NA	1.14	0.52	1.20	-0.06	0.95
Esox americanus	-1.15	0.20	-0.05	-0.42	-0.07	0.76	-0.45	0.42	-0.03	-0.38	0.06	0.03	-0.06	-0.20	-0.08	1.56	-1.73	0.68
Esox lucius	-14.81	0.71	-1.72	0.39	-0.97	1.49	-1.16	-228.58	-923.08	0.17	NA	0.17	NA	-0.73	-0.33	3.22	-0.59	0.90
Etheostoma blennioides	-0.08	-0.11	-0.04	-0.35	-0.09	0.17	-0.03	-2.62	0.17	0.63	-0.16	-0.60	0.09	0.72	-0.01	0.36	-0.04	0.75
Etheostoma caeruleum	-0.16	-0.06	NA	0.24	0.04	0.00	NA	-3.00	0.21	0.97	-0.20	-0.91	0.15	1.00	0.04	-0.94	0.04	0.79
Etheostoma camurum	-35.87	0.76	-0.79	-0.19	NA	-1.78	-2.09	-469.32	-1826.28	3.09	-1.01	-3.40	0.83	2.73	-0.30	0.92	-0.07	0.96
Etheostoma flabellare	0.01	-0.09	-0.03	-0.43	-0.11	-0.51	0.02	-1.18	0.09	0.33	-0.02	-0.93	0.11	0.52	-0.18	-1.62	0.09	0.79
Etheostoma nigrum	1.22	0.22	-0.14	-0.51	-0.14	-0.26	0.01	-0.35	0.03	0.05	NA	0.05	NA	0.09	-0.06	-1.36	0.07	0.70
Etheostoma tippencanoe	-7.40	0.32	-1.45	-0.40	0.08	-3.12	0.12	-4.10	NA	4.06	-1.94	-2.74	0.65	1.39	-0.35	6.58	-2.26	0.98
Etheostoma variatum	-4.28	-0.02	NA	-0.30	-0.03	-1.99	0.14	-17.96	-179.44	0.35	NA	-0.60	-0.16	1.87	0.39	0.37	-0.03	0.92
Etheostoma zonale	-1.71	0.15	-0.04	-0.16	-0.05	0.00	NA	-0.94	-0.19	0.91	-0.23	-1.11	0.20	0.94	0.12	0.82	-0.05	0.80
Fundulus notatus	-2.29	0.57	-0.06	-0.38	-0.22	0.33	-0.01	-4.16	0.34	1.63	-0.54	-0.49	0.10	-0.62	0.01	1.88	-2.00	0.82
Hybopsis amblops	-4.56	-0.65	-0.31	-0.03	NA	-0.63	-0.18	-3.12	-30.32	1.08	-0.21	-1.94	-0.26	1.44	-0.20	0.72	-0.05	0.87
Hypentelium nigricans	0.42	-0.07	-0.04	0.07	-0.03	-0.55	0.02	-0.68	0.05	0.08	NA	-0.37	0.06	0.99	-0.05	4.29	-0.27	0.84
Ictalurus punctatus	-1.71	0.90	-0.21	-0.32	-0.12	0.51	-0.20	-0.33	NA	0.33	-0.15	-0.28	NA	0.45	-0.01	4.01	-0.23	0.91
Ictiobus bubalus	-4.17	0.45	-0.11	-0.93	-0.43	-1.20	-0.06	-28.00	-266.78	-0.52	-0.65	-0.14	NA	1.82	-0.27	0.74	-0.03	0.96
Labidesthes sicculus	-2.98	0.58	-0.17	-0.15	NA	0.05	NA	-0.47	-0.62	0.46	-0.65	-1.42	0.30	0.46	-0.02	0.60	-0.04	0.78
Lampetra aepyptera	-3.73	-0.21	NA	-0.31	-0.02	-1.08	-0.03	-1.43	0.12	-0.10	-0.34	-1.62	0.27	0.17	-0.19	-0.37	NA	0.87
Lepisosteus osseus	-4.44	-0.05	NA	0.08	NA	-1.35	0.07	-6.14	-89.24	-0.31	-0.55	-0.12	NA	1.30	0.20	0.96	-0.04	0.90
Lepomis cyanellus	1.39	0.29	-0.09	-0.22	-0.04	0.60	-0.02	0.02	NA	-0.05	NA	0.22	-0.03	0.19	-0.06	-0.37	0.02	0.65
Lepomis gibbosus	-3.31	0.05	NA	0.33	0.03	0.66	-0.18	-9.25	0.73	-1.84	0.07	1.31	-0.27	-0.03	NA	0.57	-0.19	0.78
Lepomis gulosus	-2.68	0.43	-0.31	-0.16	0.02	-0.23	-0.10	0.09	NA	-1.46	-0.32	0.81	-0.07	-0.31	-0.09	2.29	-2.69	0.76
Lepomis humilis	-3.27	0.72	-0.08	-0.43	-0.14	0.99	-0.16	-3.90	-7.10	0.95	-0.35	-0.47	0.14	-0.12	-0.11	0.88	-0.05	0.81
Lepomis macrochirus	0.81	0.14	-0.06	0.05	NA	0.09	0.00	0.03	NA	-0.11	NA	0.11	NA	0.39	0.08	0.70	-0.05	0.64
Lepomis megalotis	-0.76	0.43	-0.07	-0.59	-0.17	-0.11	0.01	-1.08	-0.03	0.78	-0.14	-0.83	0.16	0.19	-0.05	1.17	-0.24	0.72
Lepomis microlophus	-3.51	-0.22	NA	-0.06	NA	0.80	-0.77	0.74	-0.20	-1.20	-0.53	-0.52	0.08	0.06	NA	0.68	-0.03	0.79

Luxilus chrysocephalus	0.48	0.30	-0.07	-0.02	-0.04	-0.33	0.02	-0.26	0.03	1.07	-0.25	-1.23	0.21	0.53	-0.09	-0.36	0.02	0.74
Luxilus cornutus	-1.87	0.29	-0.16	0.37	0.06	0.16	-0.01	-1.83	0.14	-1.08	0.17	1.18	-0.27	0.07	0.08	-0.57	0.03	0.72
Lythrurus fasciolaris	-0.15	-0.07	NA	-0.49	-0.10	0.02	NA	3.14	-16.82	1.50	-0.45	-2.17	0.35	0.34	-0.13	1.52	-8.86	0.79
Lythrurus umbratilis	-1.42	0.76	-0.22	-0.51	-0.19	0.16	0.00	-1.44	-5.85	0.46	0.01	0.18	-0.14	-0.59	-0.12	3.48	-3.49	0.75
Micropterus dolomieux	0.24	-0.12	-0.04	0.08	NA	-0.04	NA	-1.55	0.07	0.79	-0.16	-0.91	0.19	0.71	-0.07	8.26	-0.54	0.87
Micropterus punctulatus	-2.23	0.60	-0.29	-1.06	-0.21	-0.73	0.03	-0.27	-0.03	-0.12	-0.22	-0.28	0.09	0.82	-0.22	1.05	-0.06	0.84
Micropterus salmoides	-0.13	0.02	-0.04	0.02	NA	0.41	-0.11	-0.28	0.03	-0.29	0.03	0.24	-0.03	0.14	0.00	0.04	NA	0.61
Minytrema melanops	-1.88	0.22	-0.22	-0.43	-0.09	0.57	-0.60	0.00	NA	0.07	NA	0.10	NA	-0.02	-0.16	2.66	-0.85	0.79
Morone chrysops	-16.61	0.43	-0.17	-0.30	-0.05	-0.25	NA	-154.06	-978.22	-0.81	-0.90	-0.75	-0.92	1.75	-0.31	0.39	-0.02	0.86
Moxostoma anisurum	-2.31	0.70	-0.26	-0.67	-0.20	-0.74	0.03	0.08	-2.97	0.07	-0.11	0.07	NA	0.65	-0.17	3.22	-0.19	0.91
Moxostoma breviceps	-3.15	-0.78	0.08	-1.35	-0.23	-0.28	NA	-8.83	-162.14	-0.40	-2.16	-1.54	0.56	3.49	-0.82	0.96	-0.05	0.97
Moxostoma carinatum	-9.36	1.11	-2.10	0.56	0.10	-0.09	NA	-77.62	-639.43	0.35	-0.06	0.36	NA	1.88	-0.51	1.15	-0.05	0.92
Moxostoma duquesnei	-1.44	0.00	NA	-0.04	NA	-1.00	0.04	3.97	-16.85	1.15	-0.57	-1.76	0.49	1.14	0.12	3.34	-0.72	0.90
Moxostoma erythrurum	-0.27	0.01	-0.04	-0.68	-0.11	-0.25	0.01	-0.90	0.07	0.69	-0.29	-1.02	0.20	0.61	0.00	5.53	-0.34	0.87
Moxostoma macrolepidotum	-22.51	1.00	-0.21	0.40	0.09	-1.58	0.36	-320.48	-1310.36	0.32	-0.11	0.21	NA	0.32	-0.24	2.35	-0.29	0.90
Nocomis biguttatus	-3.20	1.16	-0.41	0.54	0.04	0.63	-0.87	-3.62	NA	1.59	-0.35	-0.94	0.14	0.33	0.14	-0.16	-6.03	0.78
Nocomis micropogon	-4.37	-0.23	-0.02	0.38	0.10	-0.21	-0.44	-5.81	-7.08	-0.98	-0.43	0.27	NA	1.57	0.00	1.65	-0.49	0.89
Notemigonus crysoleucas	-2.27	0.25	-0.05	0.09	NA	0.56	-0.14	0.00	NA	-0.71	0.09	0.58	-0.06	-0.55	-0.12	0.20	-0.48	0.70
Notropis atherinoides	-2.07	1.30	-1.03	-0.41	-0.53	-0.11	NA	3.51	-7.65	-0.28	-0.39	-0.06	-0.36	0.67	0.11	0.91	-0.05	0.86
Notropis buccatus	-0.41	0.10	-0.07	-0.35	-0.08	-0.73	0.11	-0.56	0.04	0.22	-0.08	-0.41	0.09	0.05	-0.07	-1.22	-0.13	0.70
Notropis photogenis	-1.01	0.05	NA	-0.25	-0.08	-0.37	0.02	3.09	-18.61	1.09	-0.26	-1.31	0.24	1.22	0.04	0.62	-0.06	0.83
Notropis rubellus	-2.72	0.42	-0.14	-0.34	-0.11	-0.75	0.03	-2.65	-3.64	0.32	-0.19	-0.21	0.17	1.11	0.19	1.09	-0.23	0.83
Notropis stramineus	-0.81	0.23	-0.09	-0.09	-0.02	-0.18	0.01	-0.64	0.01	0.43	-0.15	-0.38	0.11	0.45	0.07	1.49	-0.09	0.74
Notropis volucellus	-3.21	0.54	-0.20	-0.20	-0.05	-0.21	NA	-1.03	-4.85	0.06	NA	-0.63	-0.11	1.12	0.04	0.79	-0.05	0.85
Noturus flavus	-2.25	0.16	-0.04	-0.08	-0.07	0.10	NA	-0.58	-0.01	0.55	-0.28	-0.63	0.11	1.25	0.04	1.96	-0.51	0.86
Noturus miurus	-3.78	-0.27	-0.29	0.10	NA	-0.88	-0.07	-1.27	-7.61	1.78	-0.65	-3.38	0.43	0.47	-0.27	3.72	-1.78	0.88
Perca flavescens	-4.27	-0.52	-0.11	0.16	-0.07	1.59	-1.01	-10.09	0.75	-0.89	0.02	0.09	NA	-0.23	0.03	1.89	-0.35	0.80
Percina caprodes	-1.00	0.11	-0.05	-0.43	-0.12	0.41	-0.09	-1.26	0.10	0.68	-0.14	-0.81	0.14	0.76	0.01	2.42	-0.15	0.83
Percina maculata	-0.84	0.51	-0.15	-0.23	-0.09	0.16	-0.09	-0.64	-0.01	0.11	NA	-0.22	-0.02	-0.09	-0.16	1.50	-0.64	0.67
Percina phoxocephala	-4.73	-0.09	NA	-1.03	-0.51	-1.05	0.14	-32.30	-266.55	-0.32	NA	0.03	NA	2.10	-0.34	0.57	-0.03	0.95
Percina sciera	-3.98	1.93	-0.71	-0.21	-2.50	-3.53	-2.52	-15.94	-190.39	-0.17	NA	-0.05	NA	0.54	-0.32	2.71	-0.53	0.92
Percopsis omiscomaycus	-2.89	0.33	-0.11	-0.28	-0.09	-0.44	-0.07	-5.20	-58.05	-0.62	0.06	-0.08	NA	0.12	-0.27	0.66	-0.11	0.72
Phenacobius mirabilis	-2.19	0.72	-0.12	0.41	-0.65	-0.14	0.01	-0.06	-6.16	0.50	-0.14	0.45	-0.03	0.09	NA	0.68	-0.04	0.75
Pimephales notatus	2.01	0.06	-0.06	0.13	0.00	0.14	NA	-0.60	0.05	0.16	-0.07	-0.07	NA	0.29	0.11	0.56	-0.05	0.69
Pimephales promelas	-1.75	0.37	-0.06	0.43	0.07	0.34	-0.01	-0.23	0.01	0.33	-0.03	0.19	-0.02	-0.47	0.00	-0.54	0.04	0.73
Pimephales vigilax	-5.87	0.04	NA	-1.06	-0.41	0.40	-1.27	-5.34	-97.65	-0.78	-0.38	-0.13	NA	1.61	-0.33	1.25	-0.07	0.96
Pomoxis annularis	-1.83	0.79	-0.24	-0.33	-0.13	1.02	-0.47	0.25	-0.02	0.02	-0.19	0.03	NA	0.25	-0.28	0.48	-0.02	0.75
Pomoxis nigromaculatus	-2.62	0.64	-0.50	0.31	-0.02	0.29	-0.02	-0.20	NA	-1.08	-1.14	0.18	NA	0.49	0.01	0.47	-0.02	0.81
Pylodictis olivaris	-4.24	0.86	-0.34	-0.68	-0.17	-3.09	-1.40	-49.85	-365.61	-0.17	-0.82	-0.87	0.42	1.28	-0.31	2.30	-0.13	0.96
Rhinichthys obtusus	-0.68	-0.56	0.04	0.60	0.09	-0.16	0.01	0.20	0.01	0.12	0.03	-0.24	0.05	0.39	-0.13	-5.13	0.32	0.79
Sander canadensis	-3.10	0.90	-0.53	-0.56	-0.45	-2.97	0.22	-28.39	-247.71	0.25	-0.49	-0.10	-0.57	1.16	-0.26	1.08	-0.08	0.93
Semotilus atromaculatus	1.95	-0.39	-0.01	0.23	0.03	0.09	0.15	0.30	0.02	0.58	0.03	-0.45	0.03	0.06	-0.11	-3.93	0.23	0.89
Umbra limi	-3.15	-0.40	-0.07	-0.28	-0.27	0.37	-0.04	-1.58	-19.07	-0.85	0.15	0.51	-0.28	-0.36	-0.04	-4.62	-14.09	0.76

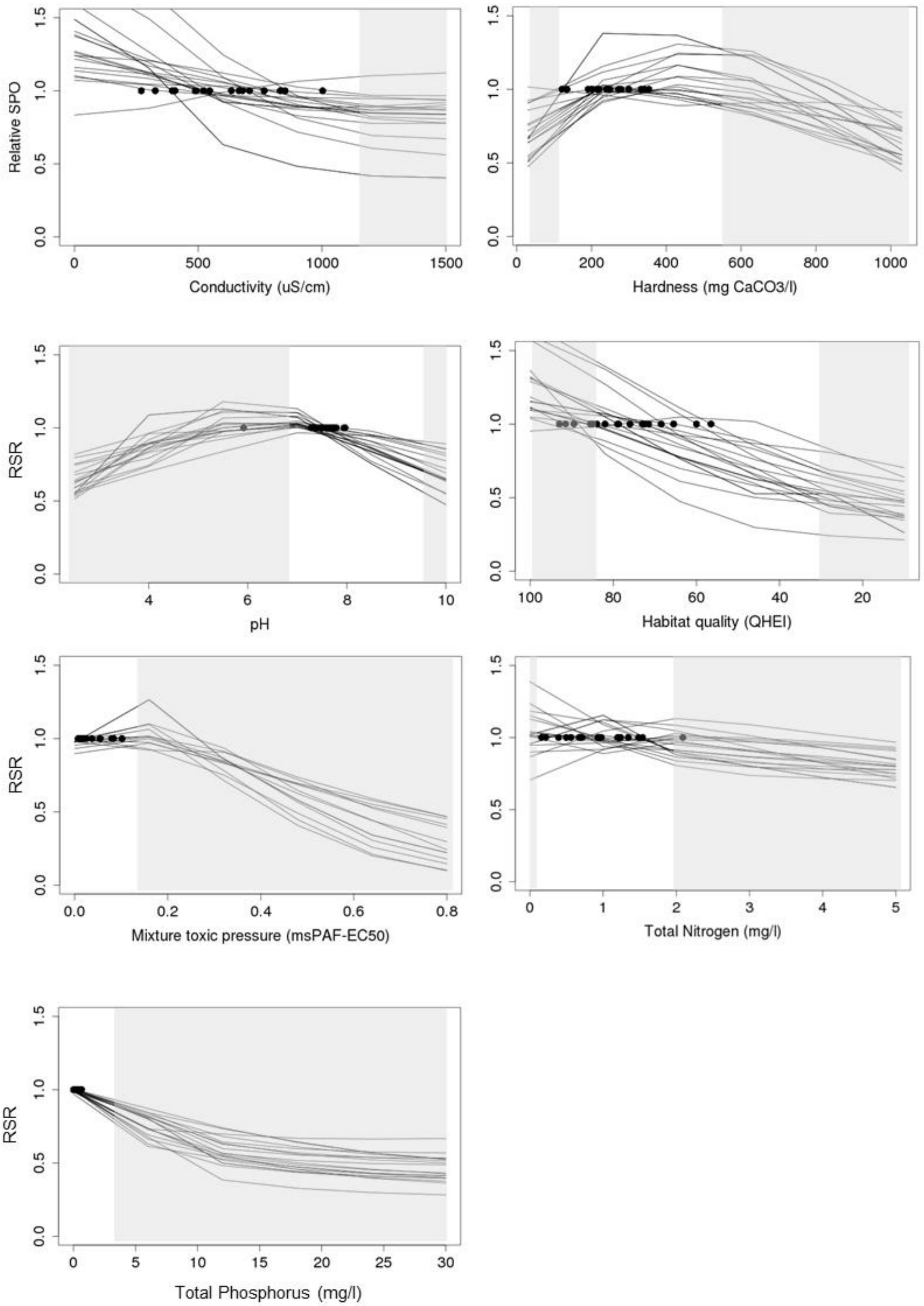

Fig S2. Modelled stressor-response relationships for Ohio fish species assemblages for the selected 18 reference sites, showing the response of relative species richness (RSR) to conductivity, hardness, pH, QHEI, mixture toxic pressure, Total N and Total P. Values of RSR higher than 1.0 indicate an

increase compared to the situation observed at the reference site and vice versa. The 'points of departure' – RSR at reference conditions – are shown as blue dots. The white areas represent 90% of the stressor-level variability in the monitoring data of Ohio. For the RSR in this figure only the SDMs with an AUC higher than 0.7 are used.

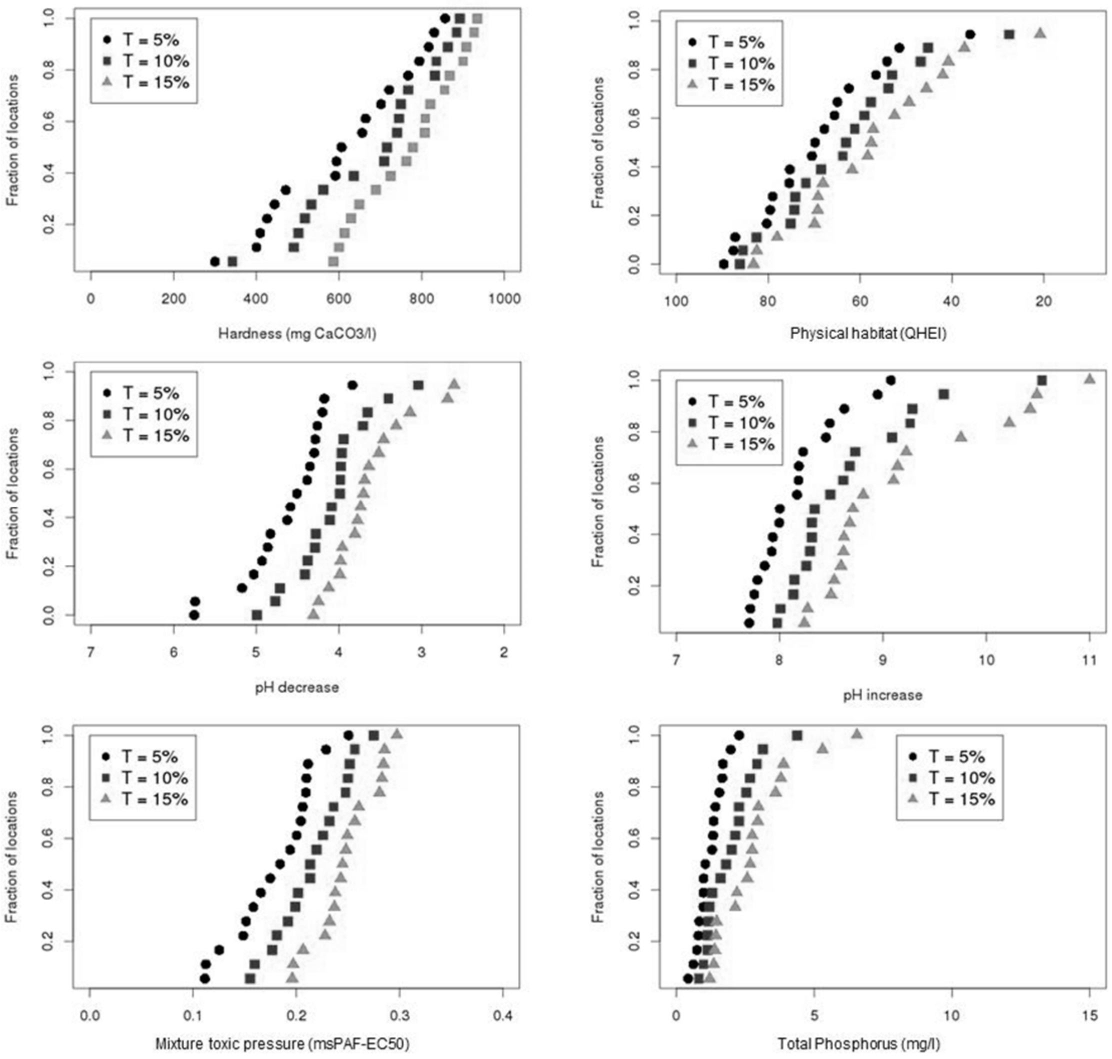

Fig. S3. Example EVDs with different choices for a selected Threshold T on the stressor-response models of Panel A in Figure 1 (main text).

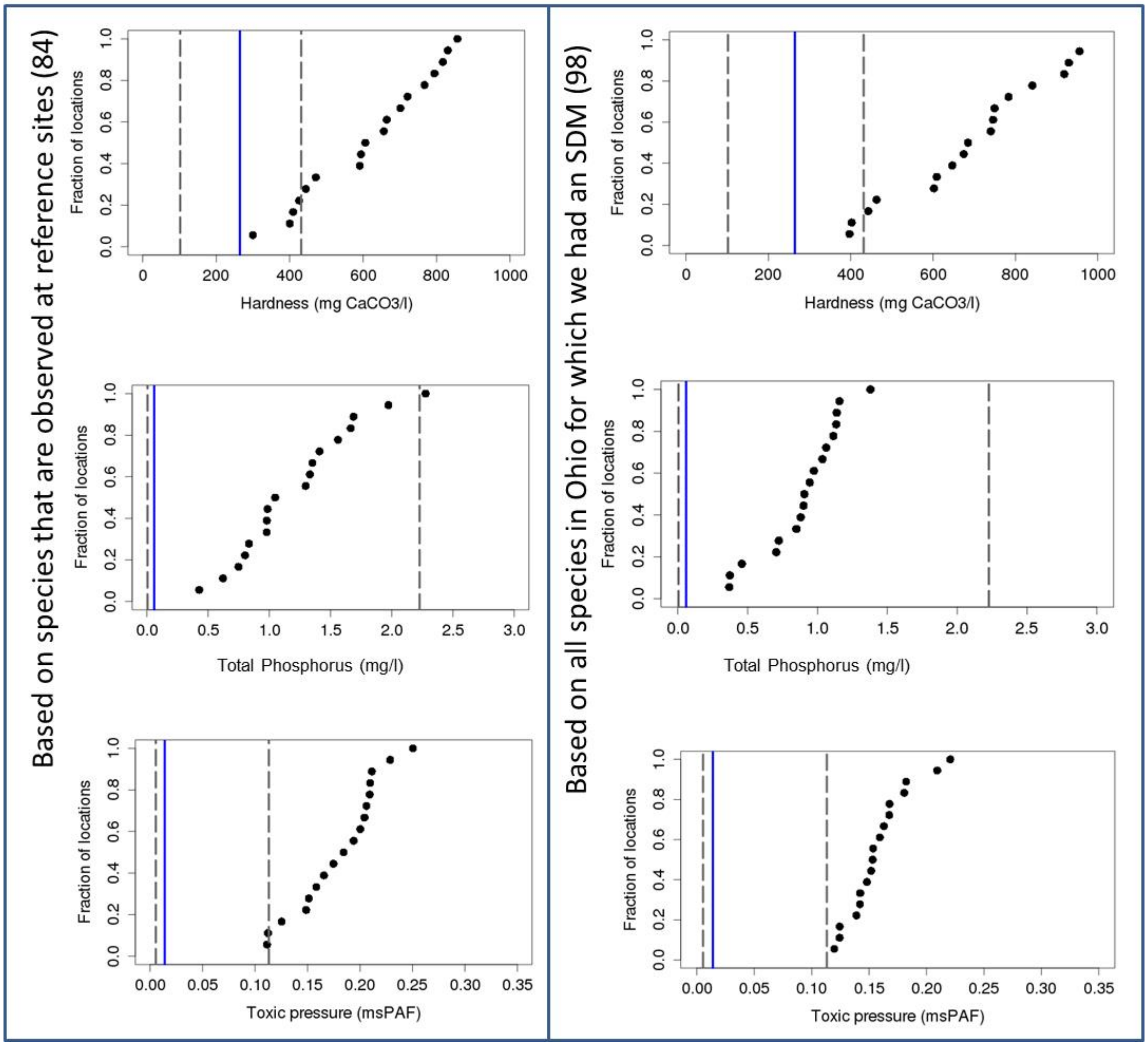

Fig. S4a. Comparison between Ecosystem Vulnerability Distributions (EVDs) derived with all species observed at the reference sites for which the database contained more the 20 data points (left panel; n = 84) and EVDs derived with all species observed in Ohio with more than 20 data points in the database (right panel; n = 98). The median stressor values across Ohio (1,826 sites) are indicated with a vertical blue line. The areas between the two dotted lines contain 90% of the stressor-level variability in the monitoring data.

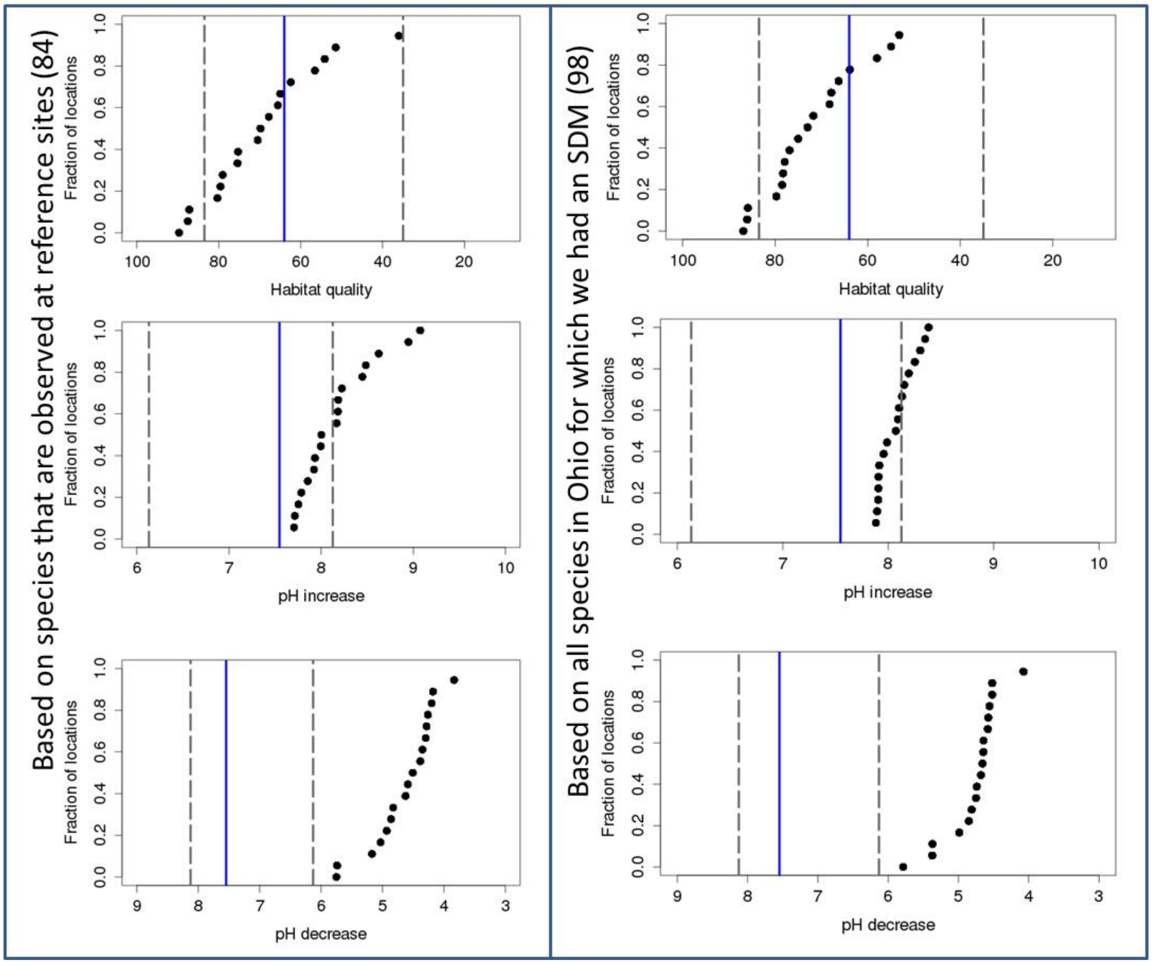

Fig. S4b. Comparison between Ecosystem Vulnerability Distributions (EVDs) derived with all species observed at the reference sites for which the database contained more the 20 data points (left panel; n = 84) and EVDs derived with all species observed in Ohio with more than 20 data points in the database (right panel; n = 98). The median stressor values across Ohio (1,826 sites) are indicated with a vertical blue line. The areas between the two dotted lines contain 90% of the stressor-level variability in the monitoring data.

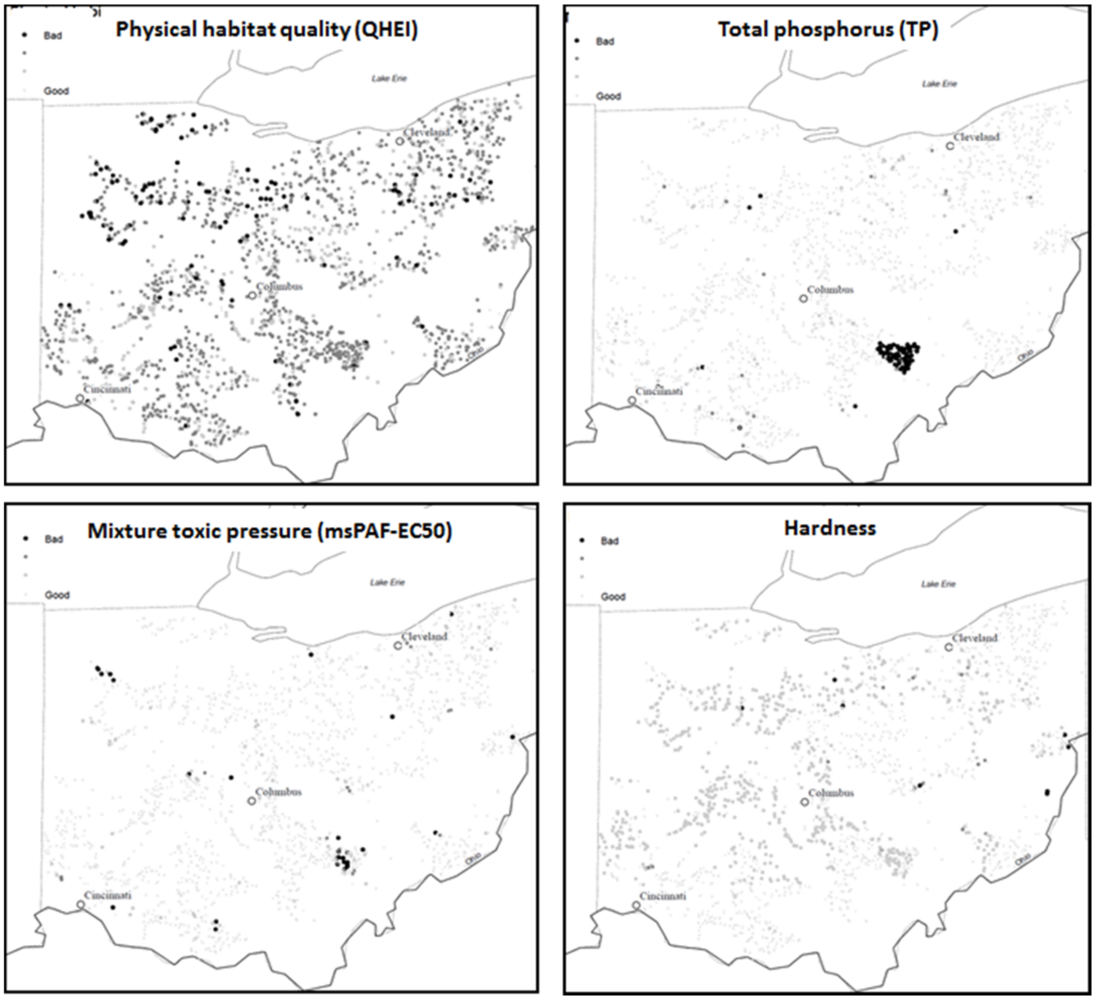

Fig. S5. Examples of the spatial presentation of the identification and ranking of stressor results (Fig. 4 main text). Back dots: locations exceeding the EVD-range defined with T=5% species loss and the 18 selected reference sites. Lighter grey and smaller dots are locations in and below the EVD range. Examples of spatial patterns are:

- upper left: physical habitat quality (QHEI). This variable was characterized by a high rank on the impact scoring at T=5% species loss; the map shows an associated wide widespread occurrence of sites where the stressor level overlays the associated EVD;
- upper right: total phosphorus (TP). This variable was characterized by a high rank of impacts >5% species loss, but a narrow band of such exceedances. The high impacts occur in the south-east, which is an area downstream of a mining area.
- lower left: mixture toxic pressure ($msPAF_{EC50}$). The ranking results were relatively similar to those of total phosphorus, but at with lower frequency. For the whole data set, mixture toxic pressure and total phosphorus had a low variance inflation factor, suggestion no collinearity across Ohio. However, the stressor identification and ranking (again) pointed at high mixture stress on species assemblages the south-eastern region, known for its historical metal mining activities. The mixture toxic pressure was defined for a large part by monitored metal concentrations.
- lower right: hardness. This variable is again characterized by a widespread expected impact, at lower rates than QHEI. This pattern is reflected in the pattern of widespread moderate exceedances shown on the map.

The maps were produced using ArcGIS version 10.2: http://resources.arcgis.com/en/help/quick-start-guides/10.2/
